# Finding Nemo’s Genes: A chromosome‐scale reference assembly of the genome of the orange clownfish *Amphiprion percula*

**DOI:** 10.1111/1755-0998.12939

**Published:** 2018-09-10

**Authors:** Robert Lehmann, Damien J. Lightfoot, Celia Schunter, Craig T. Michell, Hajime Ohyanagi, Katsuhiko Mineta, Sylvain Foret, Michael L. Berumen, David J. Miller, Manuel Aranda, Takashi Gojobori, Philip L. Munday, Timothy Ravasi

**Affiliations:** ^1^ KAUST Environmental Epigenetic Program, Division of Biological and Environmental Sciences and Engineering King Abdullah University of Science and Technology Thuwal Saudi Arabia; ^2^ Red Sea Research Center, Division of Biological and Environmental Sciences and Engineering King Abdullah University of Science and Technology Thuwal Saudi Arabia; ^3^ Computational Bioscience Research Center King Abdullah University of Science and Technology Thuwal Saudi Arabia; ^4^ ARC Centre of Excellence for Coral Reef Studies James Cook University Townsville Queensland Australia; ^5^ Evolution, Ecology and Genetics, Research School of Biology Australian National University Canberra Australian Capital Territory Australia; ^6^ Present address: Department of Environmental and Biological Sciences University of Eastern Finland Joensuu Finland

**Keywords:** *Amphiprion percula*, chromosome‐scale assembly, coral reef fish, fish genomics, functional genomics, Nemo, orange clownfish

## Abstract

The iconic orange clownfish, *Amphiprion percula*, is a model organism for studying the ecology and evolution of reef fishes, including patterns of population connectivity, sex change, social organization, habitat selection and adaptation to climate change. Notably, the orange clownfish is the only reef fish for which a complete larval dispersal kernel has been established and was the first fish species for which it was demonstrated that antipredator responses of reef fishes could be impaired by ocean acidification. Despite its importance, molecular resources for this species remain scarce and until now it lacked a reference genome assembly. Here, we present a de novo chromosome‐scale assembly of the genome of the orange clownfish *Amphiprion percula*. We utilized single‐molecule real‐time sequencing technology from Pacific Biosciences to produce an initial polished assembly comprised of 1,414 contigs, with a contig N50 length of 1.86 Mb. Using Hi‐C‐based chromatin contact maps, 98% of the genome assembly were placed into 24 chromosomes, resulting in a final assembly of 908.8 Mb in length with contig and scaffold N50s of 3.12 and 38.4 Mb, respectively. This makes it one of the most contiguous and complete fish genome assemblies currently available. The genome was annotated with 26,597 protein‐coding genes and contains 96% of the core set of conserved actinopterygian orthologs. The availability of this reference genome assembly as a community resource will further strengthen the role of the orange clownfish as a model species for research on the ecology and evolution of reef fishes.

## 1 INTRODUCTION

The orange clownfish, *Amphiprion percula*, which was immortalized in the film “Finding Nemo,” is arguably the most recognized fish on Earth. It is also one of the most important species for studying the ecology and evolution of coral reef fishes. The orange clownfish is used as a model species to study patterns and processes of social organization (Buston & Wong, 2014; Buston, Bogdanowicz, Wong, & Harrison, 2007; Wong, Uppaluri, Medina, Seymour, & Buston, 2016), sex change (Buston, 2003), mutualism (Schmiege, D'Aloia, & Buston, 2017), habitat selection (Dixson et al., 2008; Elliott & Mariscal, 2001; Scott & Dixson, 2016), lifespan (Buston & García, 2007) and predator–prey interactions (Dixson, 2012; Manassa, Dixson, McCormick, & Chivers, 2013). It has been central to ground‐breaking research into the scale of larval dispersal and population connectivity in marine fishes (Almany et al., 2017; Pinsky et al., 2017; Planes, Jones, & Thorrold, 2009; Salles et al., 2016) and how this influences the efficacy of marine protected areas (Berumen et al., 2012; Planes et al., 2009). It is also used to study the ecological effects of environmental disturbances in marine ecosystems (Hess, Wenger, Ainsworth, & Rummer, 2015; Wenger et al., 2014), including climate change (McLeod et al., 2013; Saenz‐Agudelo, Jones, Thorrold, & Planes, 2011) and ocean acidification (Dixson, Munday, & Jones, 2010; Jarrold, Humphrey, McCormick, & Munday, 2017; Munday et al., 2009; Simpson et al., 2011). Perhaps more than any other species, the orange clownfish has become a mainstay of research into the chemical, molecular, behavioural, population, conservation and climate change ecology of marine fishes.

The orange clownfish is one of 30 species of anemonefishes belonging to the subfamily Amphiprioninae within the family Pomacentridae (damselfishes). The two clownfishes, *A. percula* (orange clownfish or clown anemonefish) and *A. ocellaris* (false clownfish or western clown anemonefish), form a separate clade, alongside *Premnas biaculeatus*, within the Amphiprioninae (Li, Chen, Kang, & Liu, 2015; Litsios & Salamin, 2014; Litsios, Pearman, Lanterbecq, Tolou, & Salamin, 2014). The two species of clownfish are easily distinguished from other anemonefishes by their bright orange body coloration and three vertical white bars. The orange clownfish and the false clownfish have similar body coloration, but largely distinct allopatric geographical distributions (Litsios & Salamin, 2014). The orange clownfish occurs in northern Australia, including the Great Barrier Reef (GBR), and in Papua New Guinea, Solomon Islands and Vanuatu, while the false clownfish occurs in the Indo‐Malaysian region, from the Ryukyu Islands of Japan, throughout South‐East Asia and south to north‐western Australia (but not the GBR). We therefore conclude that the species depicted in the movie “Finding Nemo” must be *A. percula*.

Like all anemonefishes, the orange clownfish has a mutualistic relationship with sea anemones. Wild adults and juveniles live exclusively in association with a sea anemone, where they gain shelter from predators and benefit from food captured by the anemone (Fautin, 1991; Fautin & Allen, 1997; Mebs, 2009). In return, the sea anemone benefits by gaining protection from predators (Fautin & Allen, 1997; Holbrook & Schmitt, 2005), from supplemental nutrition from the clownfish's waste (Holbrook & Schmitt, 2005) and from increased gas exchange as a result of increased water flow provided by clownfish movement and activity (Herbert, Bröhl, Springer, & Kunzmann, 2017; Szczebak, Henry, Al‐Horani, & Chadwick, 2013). The orange clownfish associates with two species of anemone, *Stichodactyla gigantea* and *Heteractis magnifica* (Fautin & Allen, 1997). Clownfish social groups typically consist of an adult breeding pair and a variable number of smaller, size‐ranked juveniles that queue for breeding rights (Buston, 2003). The breeding female is larger than the male. If the female disappears, the male changes sex to female and the largest nonbreeder matures into a breeding male. The breeding pair lays clutches of demersal eggs in close proximity to their host anemone. Eggs hatch after 7–8 days and the larvae disperse into the open ocean for a period of 11–12 days, at which time they return to the reef and settle to an anemone.

The close association of clownfish and other anemonefishes with sea anemones makes them excellent species for studying aspects of marine mutualisms and habitat selection. The easily identified and delineated habitat they occupy, along with the ease with which the fish can be observed in nature, makes them ideal candidates for behavioural and population ecology. The unique capacity to collect juveniles immediately after they have settled to the reef from their pelagic larval phase also makes them ideally suited to testing long‐standing questions about larval dispersal and population connectivity in reef fish populations. Using molecular techniques to assign parentage between newly settled juveniles and adult anemonefishes, recent studies have been able to describe for the first time the spatial scales of dispersal in reef fish and its temporal consistency (Almany et al., 2017). The ability to map the connectivity of clownfish populations in space and time has also opened the door to addressing challenging questions about selection, fitness and adaptation in natural populations of marine fishes (Pinsky et al., 2017; Salles et al., 2016). Finally, the orange clownfish is one of the relatively few coral reef fishes that can easily be reared in captivity (Wittenrich, 2007). Consequently, it has unrivalled potential for experimental manipulation to test ecological and evolutionary questions in marine ecology (Dixson et al., 2014; Manassa et al., 2013), including the impacts of climate change and ocean acidification (Nilsson et al., 2012). Increasingly, genomewide methods are being used to test ecological and evolutionary questions and this is particularly true for coral reef species in the wake of anthropomorphic climate change and its effects on these sensitive ecosystems (Stillman & Armstrong, 2015).

To date, genome assemblies of two anemonefish, *A. frenatus* (Marcionetti, Rossier, Bertrand, Litsios, & Salamin, 2018) and *A. ocellaris* (Tan et al., 2018), have been published. Both of these were based on short‐read Illumina technology with genome scaffolding provided by shallow coverage of PacBio (Marcionetti et al., 2018) or Oxford Nanopore (Tan et al., 2018) long reads. While the use of long reads to scaffold Illumina‐based assemblies improves contiguity, both genome assemblies are highly fragmented with respective contig and scaffold N50s of 14.9 and 244.5 kb for *A. frenatus* and 323.6 and 401.7 kb for *A. ocellaris*. Here, we present a chromosome‐scale genome assembly of the orange clownfish, which was assembled using a primary PacBio long read strategy, followed by scaffolding with Hi‐C‐based chromatin contact maps. The resulting final assembly is highly contiguous with contig and scaffold N50 values of 3.12 and 38.4 Mb, respectively. This assembly will be a valuable resource for the research community and will further establish the orange clownfish as a model organism for genetic and genomic studies into ecological, evolutionary and environmental aspects of reef fishes. To facilitate the use of this resource, we have developed an integrated database, the Nemo Genome DB (www.nemogenome.org), which allows for the interrogation and mining of genomic and transcriptomic data described here.

## 2 MATERIALS AND METHODS

### 2.1 Specimen collection and DNA extraction

Adult orange clownfish breeding pairs were collected on the northern GBR in Australia. Fish were bred at the Experimental Aquarium Facility of James Cook University (JCU) and one individual offspring was sacrificed at the age of 8 months. The whole brain was excised, snap frozen and kept at −80°C until processing. High molecular weight DNA was extracted from whole brain tissue using the Qiagen Genomic‐tip 100/G extraction kit. The tissue was first homogenized in lysis buffer G2 supplemented with 200 μg/ml RNase A using sterile beads for 30 s. After homogenization, proteinase K was added and the homogenate was incubated at 50°C overnight. DNA extraction was then performed according to the manufacturer's protocol with a final elution volume of 200 μl. DNA fragment size and quality were assessed using pulsed‐field gel electrophoresis. This study was completed under JCU animal ethics permits A1961 and A2255.

### 2.2 PacBio library preparation and sequencing

For Pacific Biosciences (PacBio) long read sequencing, the extracted orange clownfish DNA was first sheared using a g‐TUBE (Covaris, MA, USA) (target size of 20 kb) and then converted into SMRTbell template libraries according to the manufacturer's protocol (Pacific Biosciences, CA, USA). Size selection was performed using BluePippin (Sage Science, MA, USA) to generate two libraries with a minimum size of 10 and 15 kb, respectively. Sequencing was performed using P6‐C4 chemistry on the PacBio RS II instrument at the King Abdullah University of Science and Technology (KAUST) Bioscience Core Laboratory (BCL) with 360 min movies. A total of 113 SMRT cells were sequenced.

### 2.3 Mitochondrial genome assembly

The published *A. percula* mitochondrial genome sequence (NC_023966) was used as a reference to filter the available PacBio reads. Only reads that mapped to the reference using bwa mem version 0.7.10 (Li, 2013) with the PacBio default parameters were retained. This yielded 274 reads with a total length of 2,431,457 bp, an N50 of 12.026 bp, and a predicted coverage of 146X. The mitochondrial reads were then assembled using the Organelle_PBA (Soorni, Haak, Zaitlin, & Bombarely, 2017) pipeline. The resulting assembly was annotated for genes using MitoAnnotator (Iwasaki et al., 2013). To confirm the species of the sampled individual, a phylogeny based on the annotated Cytochrome c oxidase subunit I gene (*COI*), Cytochrome b *(Cyt b)* and 12S rRNA was constructed. The sequence data of 11 anemonefish species (*A. akallopisos*—NC_030590, *A. bicinctus*—NC_016701, *A. clarkia*—NC_023967, *A. ephippium*—NC_030589, *A. frenatus*—NC_024840, *A. ocellaris*—NC_009065/AB979697/AB980197, *A. percula*—KJ174497/AB979450, *A. perideraion*—NC_024841, *A. polymnus*—NC_023826, *A. sebae*—NC_030591*, P. biaculeatus*—KJ833754) as well as the Indo‐Pacific sergeant (*Abudefduf vaigiensis*—NC009064) were utilized. The sequences were aligned for each gene using ClustalW version 2.1 (Stamatakis, 2006) with default parameters. Finally, a maximum‐likelihood phylogenetic tree was derived from the concatenated multiple alignments using RAxML (Larkin et al., 2007) with the GTRGAMMA model and 500 rounds of bootstrapping (parameters: ‐m GTRGAMMA ‐f a ‐N 500).

### 2.4 Genome assembly

The genome sequence was assembled from the unprocessed PacBio reads (Table S1) using the hierarchical diploid aware PacBio assembler falcon version 0.4.0 (Chin et al., 2016). To obtain the optimal assembly, different parameters were tested (Table S2) to generate 12 candidate assemblies. The contiguity of these assemblies was assessed with quast version 3.2 (Gurevich, Saveliev, Vyahhi, & Tesler, 2013), while assembly completeness was determined with busco version 2.0 (Simão, Waterhouse, Ioannidis, Kriventseva, & Zdobnov, 2015). Assembly “A7” exhibits the highest contiguity and single‐copy orthologous gene completeness and was selected for further improvement. The FALCON_Unzip algorithm was then applied to the initial A7 assembly obtain a haplotype‐resolved, phased assembly, termed “A7‐phased.” Contigs less than 20 kb in length were removed from the assembly. This phased assembly was polished with Quiver to achieve final consensus sequence accuracies comparable to Sanger sequencing (Chin et al., 2013) using default settings, which produced the “A7‐phased‐polished” assembly.

### 2.5 Genome assembly scaffolding with chromatin contact maps

The flash‐frozen brain tissue was sent to Phase Genomics (Seattle, WA, USA) for the construction chromatin contact maps. Tissue fixation, chromatin isolation, library preparation and 80‐bp paired‐end sequencing were performed by Phase Genomics. The sequencing reads were aligned to the A7‐phased‐polished version of the assembly with BWA (Li & Durbin, 2010) and uniquely mapping read pairs were retained. Contigs from the A7‐phased‐polished assembly were clustered, ordered and then oriented using Proximo (Bickhart et al., 2017; Burton et al., 2013), with settings as previously described (Peichel, Sullivan, Liachko, & White, 2017). Briefly, contigs were clustered into chromosomal groups using a hierarchical clustering algorithm based on the number of read pairs linking scaffolds, with the final number of groups specified as the number of the haploid chromosomes. The haploid chromosome number was set as 24, which is consistent with the observed haploid chromosome number of the Amphiprioninae, as published for *A. ocellaris* (Arai, Inoue, & Ida, 1976), *A. frenatus*, (Molina & Galetti, 2004; Takai & Kosuga, 2007), *A. clarkii* (Arai & Inoue, 1976; Takai & Kosuga, 2007), *A. perideraion* (Supiwong et al., 2015) and *A. polymnus* (Tanomtong et al., 2012). After clustering into chromosomal groups, the scaffolds were ordered based on Hi‐C link densities and then oriented with respect to the adjacent scaffolds using a weighted directed acyclic graph of all possible orientations based on the exact locations of the Hi‐C links between scaffolds. Gaps between contigs were represented with 100 Ns and the proximity‐guided assembly was named “A7‐PGA.” Gaps in the scaffolded assembly were subsequently closed using PBJelly from PBSuite version 15.8.24 (English et al., 2012) with the entire PacBio read dataset and blasr (Chaisson & Tesler, 2012) (parameters: ‐‐minMatch 8 ‐‐minPctIdentity 70 ‐‐bestn 1 ‐‐nCandidates 20 ‐‐maxScore −500 ‐‐nproc 32 ‐‐noSplitSubreads), to give rise to the final version of the assembly, “Nemo v1.”

### 2.6 Genome assembly validation

Genomic DNA was extracted from a second individual, and Illumina sequencing libraries were prepared using the NEBNext Ultra II DNA library prep kit for Illumina following the manufacturer's protocol. Three cycles of PCR were used to enrich the library. The sequencing libraries were sequenced on two lanes of a HiSeq 2500 at the KAUST BCL. A total of 1,199,533,204 paired reads were generated, covering approximately 181 Gb. The 151‐bp paired‐end reads were processed with Trimmomatic version 0.33 to remove adapter sequences and low‐quality stretches of nucleotides (parameters: 2:30:10 LEADING:20 TRAILING:20 SLIDINGWINDOW:4:20 MINLEN:75) (Bolger, Lohse, & Usadel, 2014).

The genome assembly size was validated by comparison to a k‐mer‐based estimate of genome size. The first half of the paired‐end reads of one sequencing lane (~25 Gb of data) was used for the k‐mer estimate of genome size. Firstly, KmerGenie (Chikhi & Medvedev, 2014) was used to determine the optimal *k*‐value for a k‐mer‐based estimation. Following that, Jellyfish version 2.2.6 (Marçais & Kingsford, 2011) was used with *k* = 71 to obtain the frequency distribution of all k‐mers with this length. The resulting distribution was analysed with Genomescope (Vurture et al., 2017) to estimate genome size, repeat content and the level of heterozygosity. To further validate the assembly, we determined the proportion of trimmed Illumina short reads that mapped to the Nemo version 1 assembly with bwa version 0.7.10 (Li & Durbin, 2010) and samtools version 1.1 (Li et al., 2009). Additionally, the completeness of the genome assembly annotation as determined by the conservation of a core set of genes was measured using busco with default parameters.

### 2.7 Repeat annotation

A species‐specific de novo repeat library was assembled by combining the results of three distinct repeat annotation methods. Firstly, RepeatModeler version 1.08 (Smit & Hubley, 2008) was used to build an initial repeat library. Secondly, we used LtrHarvest (Ellinghaus, Kurtz, & Willhoeft, 2008) and LTRdigest (Steinbiss, Willhoeft, Gremme, & Kurtz, 2009), both accessed via genometools 1.5.6 (Gremme, Steinbiss, & Kurtz, 2013), with the following parameters: ‐seed 76 ‐xdrop 7 ‐mat 2 ‐mis ‐2 ‐ins ‐3 ‐del ‐3 ‐mintsd 4 ‐maxtsd 20 ‐minlenltr 100 ‐maxlenltr 6000 ‐maxdistltr 25000 ‐mindistltr 1500 ‐similar 90. The resulting hits were filtered with LTRdigest, accepting only sequences featuring a hit to one of the hidden markov models in the GyDB 2.0 database. Thirdly, TransposonPSI version 08222010 (Haas, 2018) was used to detect sequences with similarities to known families of transposon open reading frames. To remove duplicated sequences in the combined result from all three methods, a clustering with USEARCH (Edgar, 2010) was performed requiring at least 90% sequence identity, and only cluster representatives were retained. The resulting representative sequences were classified by RepeatClassifier (part of RepeatModeler), Censor version 4.2.29 (Jurka, Klonowski, Dagman, & Pelton, 1996) and Dfam version 2.0 (Wheeler et al., 2012), and were then blasted against the Uniprot/Swissprot database (release 2017_12) to obtain a unified classification. Furthermore, these three classification methods and the blast result were used to filter out spurious matches to protein‐coding sequence. Specifically, putative repeat sequences were only retained when at least one classification method recognized the sequence as a repeat and the best match in Swissprot/Uniprot was not a protein‐coding gene (default blastx settings). Furthermore, sequences were retained if two of the three identification methods classified the sequence as repeat, but the best blast hit was not a transposable element. This de novo library was combined with the thoroughly curated zebrafish repeat library provided by Repbase version 22.05 (Bao, Kojima, & Kohany, 2015) and this combined library was employed for repeat masking in the Nemo version 1 assembly using RepeatMasker (Smit, Hubley, & Green, 2010).

### 2.8 RNA extraction, library construction, sequencing and read processing

Tissues for RNA extraction were dissected from one eight‐month‐old orange clownfish individual. RNA was extracted from skin, eye, muscle, gill, liver, kidney, gallbladder, stomach and fin tissues using the Qiagen AllPrep kit following manufacturer's instructions. Sequencing libraries were prepared using the TruSeq Stranded mRNA Library Preparation kit and 150‐bp paired‐end sequencing was performed on one lane of an Illumina HiSeq 4000 machine in the KAUST BCL. The RNA‐seq reads were trimmed with Trimmomatic version 0.33 (Bolger et al., 2014) (parameters: 2:30:10 LEADING:3 TRAILING:3 SLIDINGWINDOW:4:15 MINLEN:40) and contamination was removed with Kraken (Wood & Salzberg, 2014) by retaining only unclassified reads.

### 2.9 Genome assembly annotation

After mapping the RNA‐seq data with star version 2.5.2b (Dobin et al., 2013) to the final assembly, an ab‐initio annotation with BRAKER1 version 1.9 (Hoff, Lange, Lomsadze, Borodovsky, & Stanke, 2016) was performed. This initial annotation identified 49,881 genes. This annotation was then integrated with external evidence using the MAKER2 version 2.31.8 (Holt & Yandell, 2011) gene annotation pipeline. First, the transcriptome of the orange clownfish was provided to MAKER2 as EST evidence in two forms, a de novo assembly of the preprocessed RNA‐seq reads obtained with Trinity version 2.4.0 (Grabherr et al., 2011), and a genome‐guided assembly performed with the Hisat2 version 2.1.0/Stringtie version 1.3.3b workflow (Pertea, Kim, Pertea, Leek, & Salzberg, 2016). Second, we combined the proteomes of zebrafish (*Danio rerio*) (GCF_000002035.6_GRCz11), Nile tilapia (*Oreochromis niloticus*) (GCF_001858045.1_ASM185804v2) and bicolor damselfish (*Stegastes partitus*) (GCA_000690725.1), together with the Uniprot/Swissprot database (release 2017_12: 554,515 sequences) and the successfully detected BUSCO genes to generate a reference protein set for homology‐based gene prediction. In the initial MAKER2 run, the annotation edit distances (AED) were calculated for the BRAKER1‐obtained annotation, and only gene annotations with an AED of less than 0.1 and a corresponding protein length of greater than 50 amino acids were retained for subsequent training of the gene prediction program snap version 2013.11.29 (Korf, 2004). Similarly, the augustus version 3.2.3 (Stanke et al., 2006) gene prediction program was trained on 1,850 gene annotations that possessed: an AED score of <0.01; an initial start codon, a terminal stop codon and no in‐frame stop codons; more than one exon; and no introns greater than 10 kb. The hidden markov gene model of GeneMark version 4.32 (Ter‐Hovhannisyan, Lomsadze, Chernoff, & Borodovsky, 2008) was trained by BRAKER1. The final annotation was then obtained in the second run of MAKER2 with the trained models for snap, GeneMark and augustus. InterProScan 5 was then used to obtain the Pfam protein domain annotations for all genes. The standard gene builds were then generated. The output was filtered to include all annotated genes with evidence (AED less than 1) or with a Pfam protein domain, as recommended (Campbell, Holt, Moore, & Yandell, 2014).

### 2.10 Functional annotation

The protein sequences produced from the genome assembly annotation were aligned to the UniProtKB/Swissprot database (release 2017_12) with blastp version 2.2.29 (parameters: ‐outfmt 5 ‐evalue 1e‐3 ‐word_size 3 ‐show_gis ‐num_alignments 20 ‐max_hsps 20) and protein signatures were annotated with InterProScan 5. The results were then integrated with Blast2GO version 4.1.9 (Gotz et al., 2008).

### 2.11 Genome assembly comparisons

For genome assembly comparisons, we compared the Nemo version 1 genome assembly to the 26 previously reported fish chromosome‐scale genome assemblies (Table S3). Comparisons were made for genome assembly contiguity and completeness. Contig N50 values are reported for the scaffold‐scale versions of each assembly and are taken from the indicated publication (Table S3), database description (Table S3) or were generated with the Perl assemblathon_stats_2.pl script (Bradnam et al., 2013). Genome assembly completeness was assessed by determining the proportion of the genome size that is contained within the chromosome content of each assembly. It should be noted that this comparison is relative to the estimated genome size and not the published assembly size. The estimated genome size was taken as either the published estimated genome size in the relevant paper (Table S3) or from the Animal Genome Size Database (Gregory, 2018). Where possible, k‐mer‐derived or flow cytometry‐based estimates of genome size were used. Before calculation, we removed stretches of Ns from the genome assemblies as these are used to arbitrarily space scaffolds and do not contain actual genome information. However, this step was not possible for the Asian arowana, southern platyfish, yellowtail or croaker genomes as the chromosome‐scale assemblies have not been made publicly available. Genome assembly completeness was determined with busco (Simão et al., 2015) using the Actinopterygii set of 4,584 genes and the AUGUSTUS zebrafish gene model provided with the software.

### 2.12 Gene homology

To investigate the gene space of the orange clownfish genome assembly, we used OrthoFinder version 1.1.4 (Emms & Kelly, 2015) to identify orthologous gene relationships between the orange clownfish and four related fish species. The following four fish species were utilized in addition to the orange clownfish: Asian seabass GCF_001640805.1_ASM164080v1 (45,223 sequences), Nile tilapia GCF_001858045.1_ASM185804v2 (58,087 sequences), southern platyfish GCF_000241075.1_*Xiphophorus_maculatus*‐4.4.2 (23,478 sequences) and zebrafish GCF_000002035.6_GRCz11 (52,829 sequences). The longest isoform of each gene was utilized in the analysis, which corresponded to 25,050, 28,497, 23,043, and 32,420 sequences, respectively. 26,597 sequences were used for the orange clownfish. These protein sequences were reciprocally blasted against each other and clusters of orthologous genes were then defined using OrthoFinder with default parameters. As part of OrthoFinder, the concatenated sequences of single‐copy orthologs present in all species were then used to construct a phylogenetic tree, which was rooted using stride (Emms & Kelly, 2017).

### 2.13 Database system architecture and software

The Nemo Genome DB database (www.nemogenome.org) was implemented on a UNIX server with CentOS version 7, Apache web server and MySQL Database server. JBrowse (Buels et al., 2016) was employed to visualize the genome assembly and genomic features graphically and interactively. JavaScript was adopted to implement client‐side rich applications. The JavaScript library, jQuery (https://jquery.com), was employed. Other conventional utilities for UNIX computing were appropriately installed on the server if necessary. All of the Nemo Genome DB resources are stored on the server and are available through HTTP access.

## 3 RESULTS AND DISCUSSION

### 3.1 Sequencing and assembly of the orange clownfish genome

**Figure 1:**
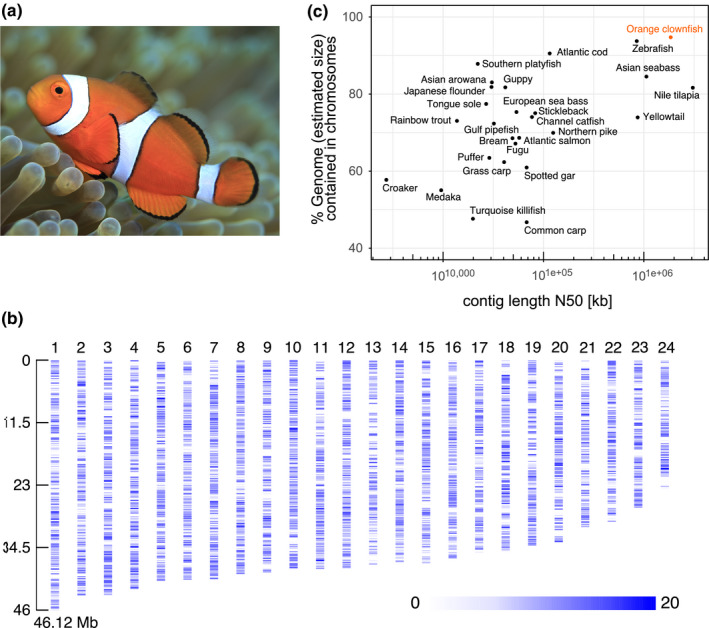
(a) The iconic orange clownfish (*A. percula*), photograph provided by Mr. Tane Sinclair‐Taylor (KAUST). (b) Gene density on the 24 chromosomes, plotted in 100 kb windows. Chromosomes are ordered by size, as indicated on the left axis in Mb. (c) Contiguity (*x*‐axis) and genome assembly completeness (*y*‐axis) of the orange clownfish, and the 26 previously published, chromosome‐scale fish genome assemblies. Details and statistics of the 27 assemblies are presented in Supporting Information Table S3 [Colour figure can be viewed at wileyonlinelibrary.com]

Genomic DNA of an individual orange clownfish (Figure 1a) was sequenced with the PacBio RS II platform to generate 1,995,360 long reads, yielding 113.8 Gb, which corresponds to a 121‐fold coverage of the genome (Table S1). After filtering with the read preassembly step of the Falcon assembler, 5,764,748 reads, covering 54.3 Gb and representing a 58‐fold coverage of the genome, were available for assembly.

To optimize the assembly parameters, we performed 12 trial assemblies using a range of parameters for different stages of the Falcon assembler (Table S2). The assembly quality was assessed by considering assembly contiguity (contig N50 and L50), total assembly size and also gene completeness (BUSCO) (Table 1). Assembly A7 exhibited the highest contig N50 (1.80 Mb), lowest contig L50 (138 contigs), lowest number of missing BUSCO genes (132) and is only slightly surpassed in the longest contig metric (15.8 Mb) by the highly similar assemblies A8 and A9 (16.5 Mb) (Table 1).

**Table 1:** Contig statistics for the preliminary candidate assemblies

Assembly	Length (Mb)	Number	N50 (Mb)	L50	Longest (Mb)	Missing genes (number, %)
A1	950.4	4,874	1.024	254	9.59	148 (3.23)
A2	945.4	4,374	1.040	251	6.67	156 (3.30)
A3	926.5	3,629	1.070	236	7.21	140 (3.05)
A4	921.8	2,829	1.380	184	8.16	134 (2.92)
A5	883.9	1,017	1.469	167	10.24	146 (3.18)
A6	902.2	2,204	1.401	174	12.38	134 (2.92)
A7	920.7	2,473	1.801	138	15.84	132 (2.88)
A8	924.6	2,629	1.742	143	16.51	139 (3.03)
A9	924.9	2,638	1.742	143	16.51	140 (3.05)
A10	917.1	2,368	1.648	140	10.21	146 (3.18)
A11	899.9	2,049	1.571	160	9.07	151 (3.29)
A12	908.8	2,086	1.602	142	10.21	143 (3.12)

Genome assemblies represent a mixture of the two possible haplotypes of a diploid individual at each locus. This collapsing of haplotypes may result in a loss of important sequence information. However, diploid aware assembly algorithms such as the Falcon_Unzip assembler are designed to detect single‐nucleotide polymorphisms (SNPs) as well as structural variations and to use this information to phase (“unzip”) heterozygous regions into distinct haplotypes (Chin et al., 2016). This procedure results in a primary assembly and a set of associated haplotype contigs (haplotigs) capturing the divergent sequences. Having established the parameter set that gave the best assembly metrics with Falcon, we used Falcon_Unzip to produce a phased assembly (“A7‐phased”) of the orange clownfish (Table 2). The phased assembly was 905.0 Mb in length with a contig N50 of 1.85 Mb. As has been seen in previous genome assembly projects (Chin et al., 2016), Falcon_Unzip produced a smaller assembly with fewer contigs than the assembly produced by Falcon (Table 2). The phased primary assembly was then polished with Quiver, which yielded an assembly (“A7‐phased‐polished”) with 1,414 contigs spanning 903.6 Mb with an N50 of 1.86 Mb (Table 2). This polishing step closed 91 gaps in the assembly and improved the N50 by approximately 14.3 kb. After polishing of the “unzipped” A7‐phased‐polished assembly, 9,971 secondary contigs were resolved, covering 340.1 Mb of the genome assembly. The contig N50 of these secondary contigs was 38.2 kb, with over 99% of them being longer than 10 kb in size. Relative to the 903.6 Mb A7‐phased‐polished primary contig assembly, the secondary contigs covered 38% of the assembly size. To the best of our knowledge, this is the first published fish genome assembly that has been resolved to the haplotype level with Falcon_Unzip.

**Table 2:** Assembly statistics of the orange clownfish genome assemblies

	A7	A7‐phased	A7‐phased‐polished	A7‐PGA	Nemo v1
Technology					
Falcon	✓	—	—	—	—
Falcon_Unzip	—	✓	✓	✓	✓
PacBio	✓	✓	✓	✓	✓
Quiver	—	—	✓	✓	✓
Hi‐C maps	—	—	—	✓	✓
PBJelly	—	—	—	—	✓
Contigs					
Length (Mb)	920.7	905.0	903.6	903.6	908.9
Number	2,473	1,505	1,414	1,414	1,045
N50 length (Mb)	1.80	1.85	1.86	1.86	3.12
L50 count	138	135	134	134	84
Longest (Mb)	15.84	15.83	15.85	15.9	16.6
No. Scaffolded	—	—	—	1,073	704
Scaffolds					
Length (Mb)	—	—	—	903.7	908.9
Number	—	—	—	365	365
N50 length (Mb)	—	—	—	38.1	38.4
L50 count	—	—	—	12	12
Longest (Mb)	—	—	—	45.8	46.1
Ns	—	—	—	104,900	32,395
Number of gaps	—	—	—	1,049	680
Chromosomes					
Length in chr (Mb)	—	—	—	885.4	890.2
% assembly in chr*	—	—	—	98.0%	97.9%
% assembly not in chr*	—	—	—	2.0%	2.1%
% of predicted genome size in chr*	—	—	—	94.3%	94.8%

*Predicted genome size is 938.88 Mb (Hardie & Hebert, 2004).

### 3.2 Scaffolding of the orange clownfish genome assembly into chromosomes

To build a chromosome‐scale reference genome assembly of the orange clownfish, chromatin contact maps were generated by Phase Genomics (Supporting Information Figure S2). Scaffolding was performed by the Proximo algorithm (Bickhart et al., 2017; Burton et al., 2013) on the A7‐phased‐polished assembly using 231 million Hi‐C‐based paired‐end reads to produce the proximity‐guided assembly “A7‐PGA” (Table 2). The contig clustering allowed the placement of 1,073 contigs into 24 scaffolds (chromosomes) with lengths ranging from 23.4 to 45.8 Mb (Tables 2 and 3). While only 76% of the contigs were assembled into chromosome clusters, this corresponds to 98% (885.4 Mb) of total assembly length and represents 95% of the estimated genome size of 938.9 Mb (Tables 2 and 3). This step substantially improved the overall assembly contiguity, raising the N50 20‐fold from 1.86 to 38.1 Mb.

**Table 3:** Chromosome metrics before and after polishing of the final assembly

Chromosome	A7‐PGA assembly		Nemo v1 assembly			
	Contigs	Length (Mb)	Contigs	Length (Mb)	Genes	Gene density (genes/Mb)
1	57	45.8	31	46.1	1,091	23.8
2	41	43.3	31	43.4	1,132	26.1
3	55	43.2	28	43.4	1,395	32.3
4	47	42.0	29	42.2	1,259	30.0
5	32	40.5	31	40.6	1,303	32.2
6	44	40.4	24	40.6	1,337	33.1
7	37	40.2	32	40.4	1,324	32.9
8	42	39.3	26	39.4	1,276	32.5
9	47	39.0	25	39.2	1,083	27.8
10	55	38.3	38	38.6	1,339	35.0
11	40	38.3	23	38.5	1,037	27.1
12	48	38.1	23	38.4	1,067	28.0
13	30	37.6	20	37.7	1,014	27.0
14	33	37.3	33	37.4	1,362	36.5
15	45	37.3	22	37.4	1,091	29.2
16	77	36.3	50	36.6	1,018	28.0
17	35	35.2	23	35.4	987	28.0
18	40	34.9	32	35.1	1,126	32.3
19	53	34.0	35	34.2	1,062	31.2
20	46	33.4	31	33.7	1,132	33.9
21	40	30.7	21	30.8	725	23.6
22	29	29.6	20	29.8	786	26.6
23	32	27.2	23	27.4	904	33.2
24	68	23.4	53	23.7	723	30.9
In chr:	1,073	885.4	704	890.2	26,309	Ave: 29.7
Not in chr:	341	18.4	341	18.8	288	15.3
Total:	1,414	903.7	1,045	908.8	26,597	Ave: 29.3

A quality score for the order and orientation of contigs within the A7‐PGA assembly was determined. This metric is based on the differential log‐likelihood of the contig orientation having produced the observed log‐likelihood, relative to its neighbours (Burton et al., 2013). The orientation of a contig was deemed to be of high quality if its placement and orientation, relative to neighbours, were 100 times more likely than alternatives (Burton et al., 2013). In A7‐PGA, the placements of 524 (37%) of the scaffolds were deemed to be of high quality, accounting for 775.5 Mb (87%) of the scaffolded chromosomes, indicating the robustness of the assembly.

A final polishing step was performed with PBJelly to generate the final Nemo v1 assembly. This polishing step closed 369 gaps, thereby improving the contig N50 by 68% and increasing the total assembly length by 5.21 Mb (Tables 2 and 3). The length of each chromosome was increased, with a range of 23.7 to 46.1 Mb (Figure 1b). Gaps were closed in each chromosome except for chromosome 14, leaving an average of only 28 gaps per chromosome (Table 3). The final assembly is 908.9 Mb in size and has contig and scaffold N50s of 3.12 and 38.4 Mb, respectively. The assembly is highly contiguous as can be observed by the fact that 50% of the genome length is contained within the largest 84 contigs. 890.2 Mb (98%) of the genome assembly size was scaffolded into 24 chromosomes, with only 18.8 Mb of the assembly failing to be grouped. The 18.8 Mb of unscaffolded assembly is comprised of 341 contigs with a contig N50 of only 57.8 kb.

### 3.3 Validation of the orange clownfish genome assembly size

The final assembly size of 908.9 Mb is consistent with the results of a Feulgen image analysis densitometry‐based study, which determined a C‐value of 0.96 pg and thus a genome size of 938.9 Mb for the orange clownfish (Hardie & Hebert, 2004). Furthermore, our assembly size is in keeping with estimates of genome size for other fish of the *Amphiprion* genus, which range from 792 to 1,193 Mb (Gregory, 2018). We additionally validated the observed assembly size by using a k‐mer‐based approach. Specifically, the k‐mer coverage and frequency distribution were plotted and fitted with a four‐component statistical model with GenomeScope (Supporting Information Figure S3a). This allowed us to generate an estimate of genome size as well as the repeat content and level of heterozygosity. However, varying the k‐value from the recommended value of 21 up to 27 yielded a corresponding increase in the estimated genome size. We therefore used KmerGenie to determine the optimal k‐mer length of 71 to capture the available sequence information. The utilization of small *k*‐values might partially explain the reported tendency of GenomeScope to underestimate the genome size (Vurture et al., 2017). The final estimate of the haploid genome length by k‐mer analysis was 906.6 Mb, with 732.8 Mb (80%) of unique sequence and a repeat content of 173.8 Mb (19%). Furthermore, the estimated heterozygosity level of 0.12% is low considering that an F1 offspring of wild caught fish was sequenced (Supporting Information Figure S3b). While the short‐read k‐mer‐based genome size estimate of 906.6 Mb matches the final assembly size of 908.9 Mb very well, the C‐value‐derived genome size estimate is slightly larger (938.9 Mb). As an additional validation of the accuracy of the genome assembly, we mapped the trimmed Illumina short reads to the Nemo version 1 assembly and observed that 95% of the reads mapped to the assembly and that 84% of the reads were properly paired.

Based on the C‐value‐derived genome size estimate, there is approximately 29.9 Mb (3.3%) of sequence length absent from our genome assembly. It seems likely that our assembly is nearly complete for the euchromatic regions of the genome given our assessment of genome size and gene content completeness. However, genomic regions such as the proximal and distal boundaries of euchromatic regions contain heterochromatic and telomeric repeats, respectively, are refractory to currently available sequencing techniques and are typically absent from genome assemblies (Bickhart et al., 2017; Hoskins et al., 2007).

### 3.4 Phylogenetic analysis of mitochondrial genes

The mitochondrial genome of *A. percula* was assembled using Organelle_PBA (Soorni et al., 2017) and mitochondrial genes were annotated using MitoAnnotator (Iwasaki et al., 2013) (Supporting Information Figure S1a). The consensus length of the mitochondrial genome is 16,638 bp, which is only 7 bp shorter than the reference sequence NC_023966. It contains 13 protein‐coding genes, 22 transfer RNA genes, one 12S and 16S ribosomal RNA, and one D‐loop control region. The sequence similarity of the complete mitogenomes between *A. percula* and *A. ocellaris* (NC_009065) is 95.5% which is consistent with previous reports (Tao, Li, Liu, & Hu, 2016). The phylogenetic analysis of the Cytochrome c oxidase subunit I (*COI*), Cytochrome b *(Cyt b)* and 12S rRNA genes from 11 anemonefish species and the Indo‐pacific sergeant revealed that the sequenced individual is most likely *A. percula* (Supporting Information Figure S1b).

### 3.5 Chromosome‐scale fish genome assembly comparisons

To date, chromosome‐scale genome assemblies have been released for 26 other fish species (Supporting Information Table S3). Here, we present the first chromosome‐scale assembly of a tropical coral reef fish, the orange clownfish. As a measure of genome assembly quality, we assessed the contiguity and completeness of these 27 chromosome‐scale genome assemblies. We investigated genome contiguity with the contig N50 metric and characterized genome completeness for each genome assembly by calculating the proportion of the estimated genome size that was assigned to chromosomes. As shown in Figure 1c, the orange clownfish genome assembly is highly contiguous, with a scaffold‐scale contig N50 of 1.86 Mb, which is only surpassed by the contig N50 of the Nile tilapia genome assembly. Interestingly, even though different assembler algorithms were utilized, the three genome assemblies based primarily on long read PacBio technology were the most contiguous, with only Nile tilapia (3.09 Mb, Canu), orange clownfish (1.86 Mb, Falcon) and Asian seabass (1.19 Mb, HGAP) genome assemblies yielding contig N50s in excess of 1 Mb.

While the use of long read sequencing technologies facilitates the production of highly contiguous genome assemblies, scaffold sizes are still much shorter than the length of the underlying chromosomes. The use of further scaffolding technologies such as genetic linkage maps, scaffolding based on synteny with genome assemblies from related organisms, as well as in vitro and in vivo Hi‐C‐based methods has allowed for the production of assemblies with chromosome‐sized scaffolds. Here, the use of Hi‐C‐based chromatin contact maps allowed for the placement of 98% of the Nemo version 1 assembly length (890.2 of 908.9 Mb) into chromosomes, yielding a final assembly with a scaffold N50 of 38.4 Mb. This corresponds to 95% of the estimated genome size (938.9 Mb), which suggests that the Nemo v1 assembly is one of the most complete fish genome assemblies published to date (Figure 1c). Only the zebrafish (94%) and Atlantic cod (91%) genome assemblies had a comparably high proportion of their estimated genome sizes scaffolded into chromosome‐length scaffolds (Figure 1c). It is likely that the use of both PacBio long reads and Hi‐C‐based chromatin contact maps contributed to the very high proportion of the orange clownfish genome that we were able to both sequence and assemble into chromosomes.

**Figure 2:**
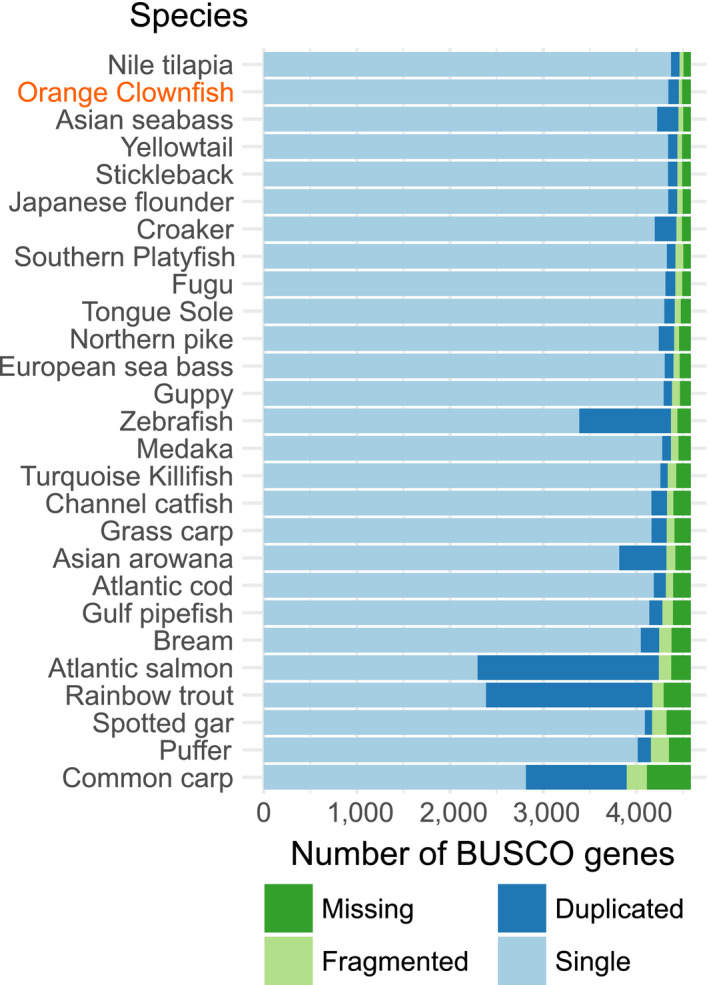
Genome assembly completeness of all published chromosome‐scale fish genome assemblies, as measured by the proportion of the BUSCO set of core genes detected in each assembly. Genome assemblies on the *y*‐axis are sorted by the sum of single copy and duplicated BUSCO genes [Colour figure can be viewed at wileyonlinelibrary.com]

While assembly contiguity is important, genome completeness with respect to gene content is also vital for producing a genome assembly that will be utilized by the research community. We evaluated the completeness of the 27 chromosome‐scale assemblies with BUSCO and the Actinopterygii lineage, which encompasses 4,584 highly conserved genes. When ranked by the total of complete (single copy and duplicate) genes, the orange clownfish assembly is the second most complete, with 4,456 (97.2%) of the orthologs identified (Figure 2). The top ranked assembly, Nile tilapia, contains only nine more of the core set of orthologs such that it contains 4,465 of the orthologs (97.4%). While the assemblies based on PacBio long read technology are again amongst the most complete, it should also be noted that most of the assemblies analysed showed a very high level of completeness.

### 3.6 Anemonefish genome assembly comparisons

Genome assemblies for *A. frenatus* (Marcionetti et al., 2018) and *A. ocellaris* (Tan et al., 2018) have been previously reported. While the *A. percula* genome assembly reported here is based on a PacBio primary assembly, the *A. frenatus* and *A. ocellaris* assemblies are based on Illumina short‐read technology, with scaffolding provided by a shallow coverage of long reads. The use of a primary PacBio assembly strategy facilitated the production of an assembly that is substantially more contiguous than the previously reported anemonefish genome assemblies (Supporting Information Table S4).

### 3.7 Genome annotation

**Figure 3:**
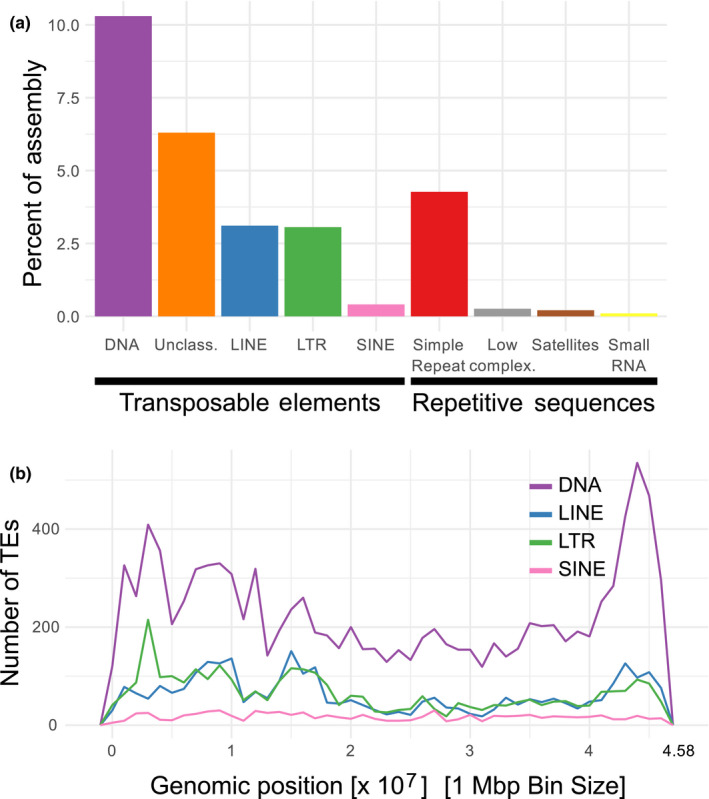
Repeat content of the orange clownfish genome assembly. (a) Repeat content of the whole genome as classified into transposable elements and repetitive sequences. (b) Spatial distribution of the four main identified classes of transposable elements on chromosome 1. Transposable element spatial distribution for chromosomes 2–24 is shown in Supporting Information Figure S4. Detailed transposable element content is shown in Supporting Information Table S4 [Colour figure can be viewed at wileyonlinelibrary.com]

To annotate repetitive sequences and transposable elements, we constructed an orange clownfish‐specific library by combining the results of Repeatmodeler, LTRharvest and TransposonPSI. Duplicate sequences were removed and false positives were identified using three classification protocols (Censor, Dfam, RepeatClassifier) as well as comparisons to Uniprot/Swissprot databases. After these filtering steps, we identified 21,644 repetitive sequences. These sequences, in combination with the zebrafish library of RepBase, were then used for genome masking with RepeatMasker. This lead to a total of 28% of the assembly being identified as repetitive (Figure 3a and Supporting Information Table S5). It was observed that there is a general trend for increased repeat density towards the ends of chromosome arms (Figure 3b and Supporting Information Figure S4). The total fraction of repetitive genomic sequence is in good agreement with other related fish species (Chalopin, Naville, Plard, Galiana, & Volff, 2015). Similarly, the high fraction of DNA transposons (~10%) is in line with DNA transposon content in other fish species (Chalopin et al., 2015) but is substantially higher than what has been reported in mammals (~3%) (Chalopin et al., 2015; Lander et al., 2001).

Following the characterization of repetitive sequences in the Nemo version 1 genome assembly, gene annotation was performed with the BRAKER1 pipeline, which trained the AUGUSTUS gene predictor with supplied RNA‐seq data, and a successive refinement with the MAKER2 pipeline. We provided BRAKER1 with mapped RNA‐seq data from 10 different tissues. This initial annotation comprised 49,881 genes with 55,273 transcripts. The gene finder models of SNAP and AUGUSTUS were refined based on the initial annotation, and MAKER2 was then used to improve the annotation using the new models and the available protein homology and RNA‐seq evidence. The resulting annotation contained 26,606 genes and 35,498 transcripts, which feature a low mean AED of 0.12, indicating a very good agreement with the provided evidence. After retaining only genes with evidence support (AED of less than 1) or an annotated Pfam protein domain, the filtered annotation was comprised of 26,597 genes, corresponding to 35,478 transcripts (Table 4). This result is broadly consistent with the average number of genes (23,475) found in the 22 diploid fish species considered in this study (Supporting Information Table S3). Compared to the initial annotation, genes in the final annotation are 61% longer (13,049 bp) and encode mRNAs that are 80% longer (17,727 bp). The proportion of the genome that is covered by coding sequences also increased to 8.1% in the final annotation. Together with the observed reduction in the gene number by 47%, this indicates a substantial reduction of likely false positive gene annotations of short length and/or few exons. The gene density across the 24 chromosomes of our assembly varied from 23.6 genes/Mb (chromosome 21) to 36.5 genes/Mb (chromosome 14), with a genomewide average of one gene every 29.7 Mb (Table 3). The spatial distribution of genes across all 24 chromosomes is relatively even (Figure 1b), with regions of very low gene density presumably corresponding to centromeric regions. We observed that the longest annotated gene was APERC1_00006329 (26.5 kb), which encodes the extracellular matrix protein FRAS1, while the gene coding for the longest protein sequence was APERC1_00011517, which codes for the 18,851 amino acid protein, Titin. Functional annotation was carried out using Blast2GO and yielded annotations for 22,507 genes (85%) after aligning the protein sequences to the UniProt/Swissprot database and annotating protein domains with InterProScan.

**Table 4:** Gene annotation statistics

	Initial BRAKER1	Final MAKER2
Genes	49,881	26,597
mRNAs	55,273	35,478
Exons	391,637	463,688
Introns	336,364	428,210
CDSs	55,273	35,478
Overlapping genes	2,407	1,852
Contained genes	744	463
Longest gene	264,684	264,684
Longest mRNA	264,684	264,684
Mean gene length	8,097	13,049
Mean mRNA length	9,841	17,727
% of genome covered by genes	44.4	38.2
% of genome covered by CDS	7.5	8.1
Exons per mRNA	7	13
Introns per mRNA	6	12
BUSCO		
Completeness	95.94%	96.25%
Complete	4,398	4,412
Single copy	3,588	3,888
Duplicated	810	524
Fragmented	138	96
Missing	48	76
Total	4,584	4,584

### 3.8 Identification of orange clownfish‐specific genes

**Figure 4:**
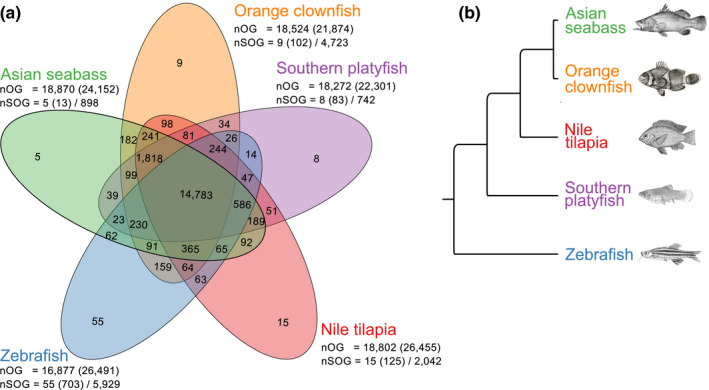
(a) The overlap of orthologous gene families of the orange clownfish, southern platyfish, Nile tilapia, zebrafish and Asian seabass. The total number of orthogroups (nOG) followed by the number of genes assigned to these groups is provided below the species name. The number of species‐specific orthogroups (nSOG) and the respective number of genes is also indicated, followed by the number of genes not assigned to any orthogroups. (b) The inferred phylogenetic tree based on the ortholog groups that contain a single gene from each species, drawings of the fish species were obtained from Wikimedia commons [Colour figure can be viewed at wileyonlinelibrary.com]

To investigate the gene space of the orange clownfish relative to other fishes, we used OrthoFinder version 1.1.4 (Emms & Kelly, 2015) to identify orthologous relationships between the protein sequences of the orange clownfish and four other fish species (Asian seabass, Nile tilapia, southern platyfish and zebrafish) from across the teleost phylogenetic tree (Betancur et al., 2013). The vast majority of sequences (89%) could be assigned to one of 19,838 orthogroups, with the remainder identified as “singlets” with no clear orthologs. We observed a high degree of overlap of protein sequence sets between all five species, with 75% of all orthogroups (14,783) shared amongst all species (Figure 4a). The proteins within these orthogroups presumably correspond to the core set of teleost genes. Of the 14,783 orthogroups with at least one sequence from each species, a subset of 8,905 orthogroups contained only a single sequence from each species. The phylogeny obtained from these single‐copy orthologous gene sequences (Figure 4b) is consistent with the known phylogenetic tree of teleost fishes (Betancur et al., 2013). Interestingly, we identified a total of 4,429 sequences that are specific to the orange clownfish, 2,293 (49%) of which possess functional annotations (Figure 4a). Future investigations will focus on the characterization of these unique genes and what roles they may play in orange clownfish phenotypic traits.

## 4 CONCLUSION

Here, we present a reference‐quality genome assembly of the iconic orange clownfish, *A. percula*. We sequenced the genome to a depth of 121X with PacBio long reads and performed a primary assembly with these reads utilizing the Falcon_Unzip algorithm. The primary assembly was polished to yield an initial assembly of 903.6 Mb with a contig N50 value of 1.86 Mb. These contigs were then assembled into chromosome‐sized scaffolds using Hi‐C chromatin contact maps, followed by gap‐filling with the PacBio reads, to produce the final reference assembly, Nemo version 1. The Nemo version 1 assembly is highly contiguous, with contig and scaffold N50s of 3.12 and 38.4 Mb, respectively. The use of Hi‐C chromatin contact maps allowed us to scaffold 890.2 Mb (98%) of the 908.2 Mb final assembly into the 24 chromosomes of the orange clownfish. An analysis of the core set of Actinopterygii genes suggests that our assembly is nearly complete, containing 97% of the core set of highly conserved genes. The Nemo version 1 assembly was annotated with 26,597 genes with an average AED score of 0.12, suggesting that most gene models are highly supported.

**Figure 5:**
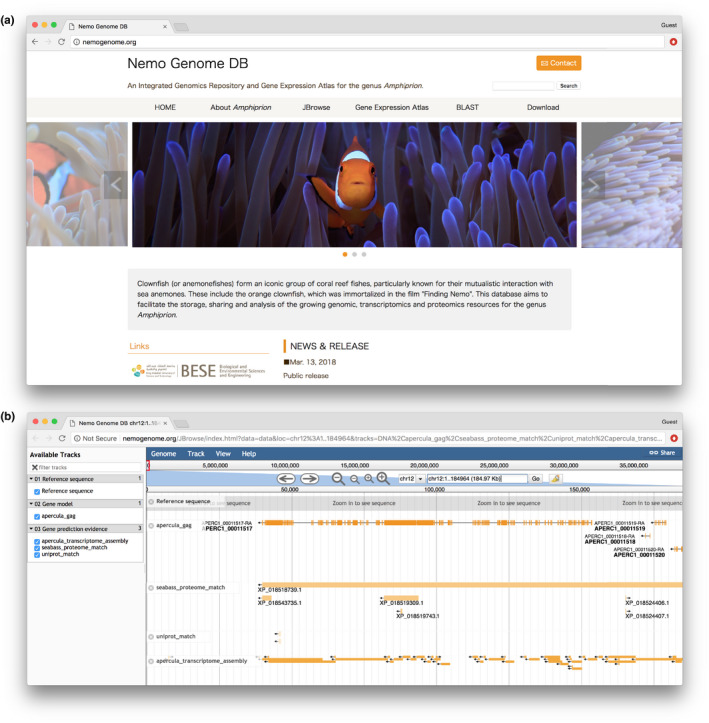
(a) Front page of the Nemo Genome DB database, which is a portal to access the data described in this manuscript and is accessible at www.nemogenome.org. (b) Genome viewer representation of the Titin gene

The high‐quality Nemo version 1 reference genome assembly described here will facilitate the use of this now genome‐enabled model species to investigate ecological, environmental and evolutionary aspects of reef fishes. To assist the research community, we have created the Nemo Genome DB database, www.nemogenome.org (Figure 5), where researchers can access, mine and visualize the genomic and transcriptomic resources of the orange clownfish.

## AUTHOR CONTRIBUTIONS

R.L. and D.J.L. designed and performed the computational analysis. R.L., T.R., C.S. and D.J.L. interpreted the results. H.O., K.M. and T.G. created the database. C.T.M. and S.F. produced sequencing libraries. R.L., D.J.L, T.R., P.L.M., M.L.B., M.A. and D.J.M. wrote the manuscript and all authors approved the final version. T.R. supervised the project.

## Data Availability

The assembled and annotated genome as well as the raw PacBio reads and Illumina reads are available at the Nemo Genome DB (https://nemogenome.org). Furthermore, the assembled nuclear and mitochondrial genome assemblies are available on GenBank as BioProject PRJNA436093 and BioSample accession SAMN08615572. Raw sequencing data described in this study are available via the NCBI Sequencing Read Archive (SRP134923).
