# Supplementary material for: Finding Nemo’s Genes: A chromosome‐scale reference assembly of the genome of the orange clownfish Amphiprion percula
Source: Mol Ecol Resour. 2018 Sep 10;19(3):570–85. doi: 10.1111/1755-0998.12939 (PMC7379943; doi:10.1111/1755-0998.12939)
Supplement: Supplementary file 1 [file MEN-19-570-s001.docx]

**Finding Nemo’s Genes: A chromosome-scale reference assembly of the genome of the orange clownfish *Amphiprion percula***

Robert Lehmann^1^, Damien J. Lightfoot^1^, Celia Schunter^1^, Craig T. Michell^2^, Hajime Ohyanagi^3^, Katsuhiko Mineta^3^, Sylvain Foret^4,5^, Michael L. Berumen^2^, David J. Miller^4^, Manuel Aranda^2^, Takashi Gojobori^3^, Philip L. Munday^4^ and Timothy Ravasi^1,*^

^1^ KAUST Environmental Epigenetic Program, Division of Biological and Environmental Sciences & Engineering, King Abdullah University of Science and Technology, Thuwal, 23955-6900, Kingdom of Saudi Arabia.

^2^ Red Sea Research Center, Division of Biological and Environmental Sciences & Engineering, King Abdullah University of Science and Technology, Thuwal, 23955-6900, Kingdom of Saudi Arabia.

^3^ Computational Bioscience Research Center, King Abdullah University of Science and Technology, Thuwal, 23955-6900, Kingdom of Saudi Arabia.

^4^ ARC Centre of Excellence for Coral Reef Studies, James Cook University, Townsville, Queensland, 4811, Australia.

^5^ Evolution, Ecology and Genetics, Research School of Biology, Australian National University, Canberra, Australian Capital Territory, 2601, Australia.

**Keywords:**

Orange Clownfish, *Amphiprion percula*, Nemo, Functional Genomics, Chromosome-Scale Assembly, Fish Genomics, Coral Reef Fish.

**Running Title:**

The Nemo Genome

**^*^Corresponding Author:**

Timothy Ravasi, Division of Biological and Environmental Sciences & Engineering, King Abdullah University of Science and Technology, Thuwal, 23955-6900, Kingdom of Saudi Arabia, [timothy.ravasi@kaust.edu.sa](mailto:timothy.ravasi@kaust.edu.sa)

**Fig. S1 (A)** Mitochondrial genome assembly of *Amphiprion percula*, annotated with MitoAnnotator. **(B)** Phylogenetic tree of Anemone fish species based on the sequence of the Cytochrome c subunit I (COI), Cytochrome b (Cyt b), and 12S rRNA genes. The sequence of the Indo-Pacific sergeant (*Abudefduf vaigiensis*) was added as outgroup. The support over 500 rounds of bootstrapping is shown on each node.

**
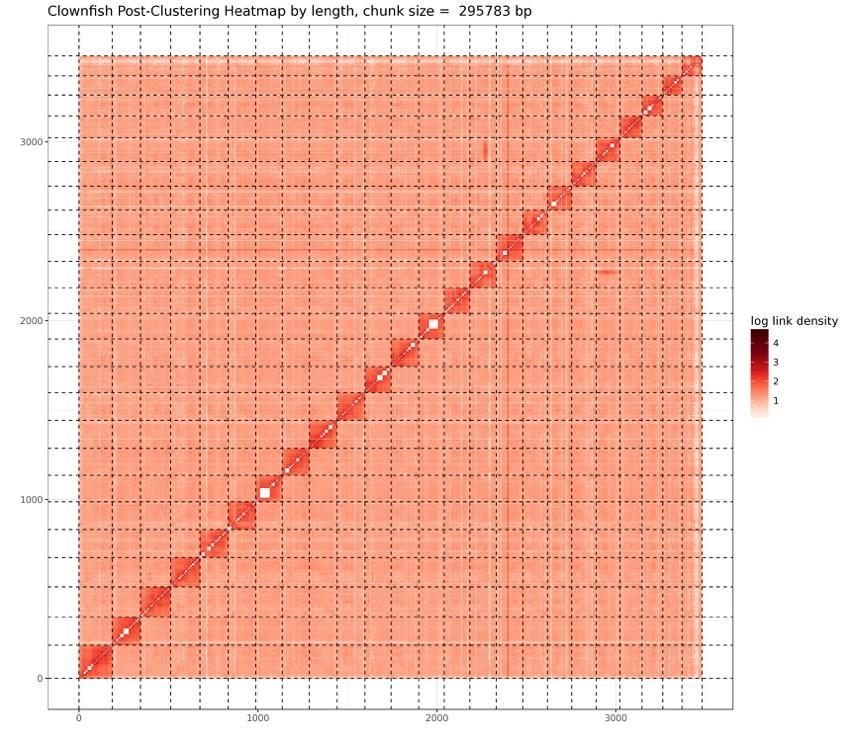
**

**Fig. S2** Hi-C interaction heat map showing the clustering of the polished primary contigs into 24 sets of chromosome-scale scaffolds.


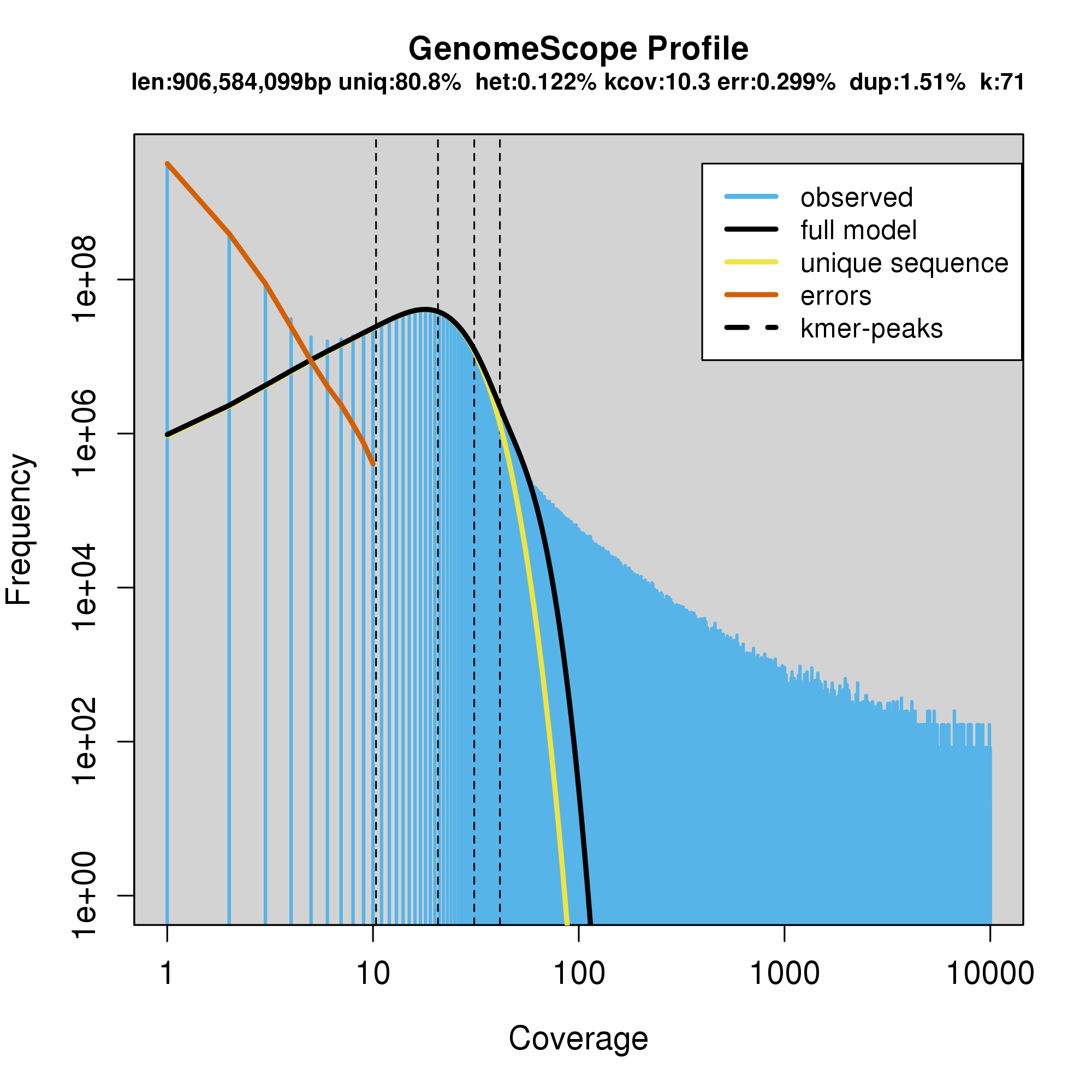


| k- value | Genome Size (Mb) | Repeat Length (Mb) | Unique Length (Mb) | Heterozygosity | Model Fit |
| --- | --- | --- | --- | --- | --- |
| 21 | 838.7 | 190.3 | 648.4 | 0.25% | 98.83% |
| 31 | 864.7 | 179.3 | 685.4 | 0.21% | 99.06% |
| 41 | 882.6 | 177.7 | 705.0 | 0.17% | 99.27% |
| 51 | 892.8 | 175.8 | 717.0 | 0.15% | 99.36% |
| 61 | 901.6 | 175.4 | 726.2 | 0.14% | 99.51% |
| 71 | 906.6 | 173.8 | 732.8 | 0.12% | 99.56% |
| 81 | 903.1 | 178.9 | 724.2 | 0.15% | 99.23% |

**Fig. S3** Estimate of the genome size based on the k-mer coverage distribution obtained with KmerGenie and k=71, which is then **(A)** fitted with a mixture model comprising four evenly spaced negative binomial distributions using GenomeScope. **(B)** The choice of k=71 optimizes the model fit of GenomeScope and yields the highest estimate for genome size and unique length with the lowest fraction of repetitive sequence and heterozygosity level.

**Fig. S4** Spatial distribution of the four main identified classes of transposable elements on chromosomes 1-24.

**Table S1** PacBio read statistics before and after filtering

|  | Raw data | Filtered data |
| --- | --- | --- |
| Read no. | 11,995,360 | 5,764,748 |
| Total (Gb) | 113.8 | 54.3 |
| Ave. length (bp) | 9,682 | 9,412 |
| N50 (bp) | 13,003 | 12,532 |
| Coverage | 121X | 58X |
| %A | 29.2 | 30.2 |
| %C | 19.3 | 19.7 |
| %G | 20.7 | 19.8 |
| %T | 30.8 | 30.3 |

* Genome coverage estimates are based on a predicted genome size of 938.88 Mb (Hardie & Hebert, 2004).

**Table S4** Anemonefish genome assembly statistics

|  | *A. percula* | *A. ocelaris*^1^ | *A. frenatus*^2^ |
| --- | --- | --- | --- |
| Assembly stats |  |  |  |
| Assembly size | 908.9 | 880.7 | 803.3 |
| Scaffolds | 365 | 6,404 | 17,801 |
| Scaffold N50 (Mb) | 38.4 | 0.401 | 0.245 |
| Longest scaffold (Mb) | 46.1 | 3.11 | 1.73 |
| Shortest scaffold (bp) | 20,004 | 672 | 1,000 |
| Ns (%) | 32,395 (0.004) | 545,178 (0.062) | 12,413,203 (1.545) |
| Annotation |  |  |  |
| Protein coding genes | 26,597 | 27,420 | 26,917 |
| Average AED score | 0.12 | 0.14 | 0.19 |
| Mean protein length | 689 | 514 | 561 |
| BUSCO |  |  |  |
| Complete (%) | 4,412 (96.2) | 4,417 (96.3) | 4,279 (93.3) |
| Complete and single copy (%) | 3,888 (84.8) | 4,269 (93.1) | 3,542 (77.3) |
| Complete and duplicated (%) | 524 (11.4) | 148 (3.2) | 737 (16.1) |
| Fragmented (%) | 96 (2.1) | 63 (1.4) | 150 (3.3) |
| Missing (%) | 76 (1.7) | 104 (2.3) | 155 (3.4) |

**^1^** Tan *et al*., 2018

^2^ Marcionetti *et al*., 2018

**Table S5** Transposable element and repeat content by family

| **Type** | **Subtype** | | **Number of hits** | **Total length (Mb)** | **Coverage (%)** |
| --- | --- | --- | --- | --- | --- |
| Transposable elements | | | | | |
| SINEs: | |  | 16,718 | 3.694 | 0.41% |
|  | | ALUs | 0 | 0 | 0.00% |
|  | | MIRs | 1,176 | 0.226 | 0.02% |
| LINEs: | |  | 66,047 | 28.233 | 3.11% |
|  | | LINE1 | 4,666 | 5.128 | 0.56% |
|  | | LINE2 | 22,304 | 11.545 | 1.27% |
|  | | L3/CR1 | 2 | 0.002 | 0.00% |
| LTR elements: | |  | 70,765 | 27.786 | 3.06% |
|  | | ERVL | 0 | 0 | 0.00% |
|  | | ERVL-MaLRs | 0 | 0 | 0.00% |
|  | | ERV_classI | 2,219 | 1.390 | 0.15% |
|  | | ERV_classII | 49 | 0.032 | 0.00% |
| DNA elements: | |  | 235,219 | 93.576 | 10.30% |
|  | | hAT-Charlie | 13,915 | 6.930 | 0.76% |
|  | | TcMar-Tigger | 4,104 | 1.094 | 0.12% |
| Unclassified: | | | 154,766 | 57.246 | 6.30% |
| Total interspersed repeats: | | |  | 210.536 | 23.16% |
| Repeats | | | | | |
| Small RNA: | | | 3,061 | 0.912 | 0.10% |
| Satellites: | | | 4,300 | 1.953 | 0.21% |
| Simple repeats: | | | 336,370 | 38.841 | 4.27% |
| Low complexity: | | | 39,390 | 2.369 | 0.26% |
| Total repeats: | | | 383,121 | 44.07 | 4.84% |

**References**

Ao, J., Mu, Y., Xiang, L.-X., Fan, D., Feng, M., Zhang, S., … Chen, X. (2015). Genome Sequencing of the Perciform Fish *Larimichthys crocea* Provides Insights into Molecular and Genetic Mechanisms of Stress Adaptation. *PLOS Genetics*, *11*(4), 1–25.

Bian, C., Hu, Y., Ravi, V., Kuznetsova, I. S., Shen, X., Mu, X., … Shi, Q. (2016). The Asian arowana (*Scleropages formosus*) genome provides new insights into the evolution of an early lineage of teleosts. *Scientific Reports*, *6*, 24501.

Braasch, I., Gehrke, A. R., Smith, J. J., Kawasaki, K., Manousaki, T., Pasquier, J., … Postlethwait, J. H. (2016). The spotted gar genome illuminates vertebrate evolution and facilitates human-to-teleost comparisons. *Nature Genetics*, *48*(4), 427–437.

Chen, S., Zhang, G., Shao, C., Huang, Q., Liu, G., Zhang, P., … Wang, J. (2014). Whole-genome sequence of a flatfish provides insights into ZW sex chromosome evolution and adaptation to a benthic lifestyle. *Nature Genetics*, *46*(3), 253–260.

Conte, M. A., Gammerdinger, W. J., Bartie, K. L., Penman, D. J., & Kocher, T. D. (2017). A high quality assembly of the Nile Tilapia (*Oreochromis niloticus*) genome reveals the structure of two sex determination regions. *BMC Genomics*, *18*, 341.

Hardie, D. C., & Hebert, P. D. (2004). Genome-size evolution in fishes. *Canadian Journal of Fisheries and Aquatic Sciences*, *61*(9), 1636–1646.

Howe, K., Clark, M. D., Torroja, C. F., Torrance, J., Berthelot, C., Muffato, M., … Stemple, D. L. (2013). The zebrafish reference genome sequence and its relationship to the human genome. *Nature*, *496*(7446).

Jaillon, O., Aury, J.-M., Brunet, F., Petit, J.-L., Stange-Thomann, N., Mauceli, E., … Roest Crollius, H. (2004). Genome duplication in the teleost fish *Tetraodon nigroviridis* reveals the early vertebrate proto-karyotype. *Nature*, *431*, 946.

Jones, F. C., Grabherr, M. G., Chan, Y. F., Russell, P., Mauceli, E., Johnson, J., … Kingsley, D. M. (2012). The genomic basis of adaptive evolution in threespine sticklebacks. *Nature*, *484*, 55.

Kai, W., Kikuchi, K., Tohari, S., Chew, A. K., Tay, A., Fujiwara, A., … Venkatesh, B. (2011). Integration of the Genetic Map and Genome Assembly of Fugu Facilitates Insights into Distinct Features of Genome Evolution in Teleosts and Mammals. *Genome Biology and Evolution*, *3*, 424–442.

Kasahara, M., Naruse, K., Sasaki, S., Nakatani, Y., Qu, W., Ahsan, B., … Kohara, Y. (2007). The medaka draft genome and insights into vertebrate genome evolution. *Nature*, *447*, 714.

Kawase, J., Aoki, J., & Araki, K. (2018). Constructing a “Chromonome” of Yellowtail (*Seriola quinqueradiata*) for Comparative Analysis of Chromosomal Rearrangements. *Journal of Genomics*, *6*, 9–19.

Künstner, A., Hoffmann, M., Fraser, B. A., Kottler, V. A., Sharma, E., Weigel, D., & Dreyer, C. (2016). The Genome of the Trinidadian Guppy, *Poecilia reticulata*, and Variation in the Guanapo Population. *PLoS ONE*, *11*(12), e0169087.

Lien, S., Koop, B. F., Sandve, S. R., Miller, J. R., Kent, M. P., Nome, T., … Davidson, W. S. (2016). The Atlantic salmon genome provides insights into rediploidization. *Nature*, *533*(7602), 200–205.

Liu, H., Chen, C., Gao, Z., Min, J., Gu, Y., Jian, J., … Wang, W. (2017). The draft genome of blunt snout bream (*Megalobrama amblycephala*) reveals the development of intermuscular bone and adaptation to herbivorous diet. *GigaScience*, *6*(7), 1–13.

Liu, Z., Liu, S., Yao, J., Bao, L., Zhang, J., Li, Y., … Waldbieser, G. C. (2016). The channel catfish genome sequence provides insights into the evolution of scale formation in teleosts. *Nature Communications*, *7*.

Peichel, C. L., Sullivan, S. T., Liachko, I., & White, M. A. (2017). Improvement of the Threespine Stickleback genome using a Hi-C-Based proximity-guided assembly. *The Journal of Heredity*, *108*(6), 693–700.

Reichwald, K., Petzold, A., Koch, P., Downie, B. R., Hartmann, N., Pietsch, S., … Platzer, M. (2015). Insights into Sex Chromosome Evolution and Aging from the Genome of a Short-Lived Fish. *Cell*, *163*(6), 1527–1538.

Rondeau, E. B., Minkley, D. R., Leong, J. S., Messmer, A. M., Jantzen, J. R., von Schalburg, K. R., … Koop, B. F. (2014). The Genome and Linkage Map of the Northern Pike (*Esox lucius*): Conserved synteny revealed between the salmonid sister group and the Neoteleostei. *PLoS ONE*, *9*(7), e102089.

Schartl, M., Walter, R. B., Shen, Y., Garcia, T., Catchen, J., Amores, A., … Warren, W. C. (2013). The genome of the platyfish, *Xiphophorus maculatus*, provides insights into evolutionary adaptation and several complex traits. *Nature Genetics*, *45*(5), 567–572.

Shao, C., Bao, B., Xie, Z., Chen, X., Li, B., Jia, X., … Chen, S. (2016). The genome and transcriptome of Japanese flounder provide insights into flatfish asymmetry. *Nature Genetics*, *49*(1), 119–124.

Small, C. M., Bassham, S., Catchen, J., Amores, A., Fuiten, A. M., Brown, R. S., … Cresko, W. A. (2016). The genome of the Gulf pipefish enables understanding of evolutionary innovations. *Genome Biology*, *17*(1), 258.

Tine, M., Kuhl, H., Gagnaire, P.-A., Louro, B., Desmarais, E., Martins, R. S. T., … Reinhardt, R. (2014). European sea bass genome and its variation provide insights into adaptation to euryhalinity and speciation. *Nature Communications*, *5*, 5770.

Tørresen, O. K., Star, B., Jentoft, S., Reinar, W. B., Grove, H., Miller, J. R., … Nederbragt, A. J. (2017). An improved genome assembly uncovers prolific tandem repeats in Atlantic cod. *BMC Genomics*, *18*(1), 95.

Vij, S., Kuhl, H., Kuznetsova, I. S., Komissarov, A., Yurchenko, A. A., Van Heusden, P., … Hall, I. (2016). Chromosomal-Level Assembly of the Asian Seabass Genome Using Long Sequence Reads and Multi-layered Scaffolding. *PLOS Genetics*, *12*(4), e1005954.

Wang, Y., Lu, Y., Zhang, Y., Ning, Z., Li, Y., Zhao, Q., … Zhu, Z. (2015). The draft genome of the grass carp (*Ctenopharyngodon idellus*) provides insights into its evolution and vegetarian adaptation. *Nature Genetics*, *47*(6), 625–631.

Xu, P., Zhang, X., Wang, X., Li, J., Liu, G., Kuang, Y., … Sun, X. (2014). Genome sequence and genetic diversity of the common carp, *Cyprinus carpio*. *Nature Genetics*, *46*, 1212.
